# Evaluation of the safety and efficacy of a Polyzene-F nanocoated coronary stent system: A systematic review and single-arm meta-analysis

**DOI:** 10.3389/fcvm.2023.1095794

**Published:** 2023-03-16

**Authors:** Jifang Bian, Rongyuan Yang, Dawei Wang, Huimin Yu, Yuan Liu, Qing Liu

**Affiliations:** ^1^ The Second Guangdong Provincial Hospital of Traditional Chinese Medicine, Guangzhou, China; ^2^ The Second Clinical School of Medicine, Guangzhou University of Chinese Medicine, Guangdong Provincial Hospital of Chinese Medicine-Zhuhai Hospital, Zhuhai, China; ^3^ The First Affiliated Hospital of Guangzhou University of Traditional Chinese Medicine, Guangdong, China; ^4^ Department of Cardiology, Guangdong Academy of Medical Sciences, Guangdong Cardiovascular Institute, Guangdong Provincial People's Hospital, Guangzhou, China

**Keywords:** coronary heart disease, Polyzene-F (PzF)-nanocoated coronary stent, safety and effectiveness, single-arm meta-analysis, COBRA and Catania systems

## Abstract

**Background:**

A stent for patients with coronary heart disease (CHD) provides a requirement for a long-term antiplatelet therapy because of the high possibility of the development of stent thrombosis. It was against this background that both Cobra and Catania Polyzene-F (PzF) stents were designed to reduce the occurrence of stent thrombosis (ST). In this study, we review the safety and effectiveness of a PzF-nanocoated stent.

**Methods:**

This systematic review with the title was registered in PROSPERO (No.398781). The inclusion criteria were including studies among patients with PzF-nanocoated coronary stents and reported target vessel failure (TVF) and ST as the outcomes, and the exclusion criteria were excluding reported patients who could not receive the adjunctive medical therapies or without the necessary endpoints. Reports about PzF-nanocoated stents were searched in PubMed, Embase, and Web of Science and other sources. Because of the existence of few reports and a lack of comparison groups, a single-arm meta-analysis was conducted in R software (v3.6.2), using a random-effects model with the generic inverse variance method. After a heterogeneity test, assessment of evidence quality was conducted by using GRADE software. A funnel plot Egger's test was performed to evaluate publication bias, and a sensitivity analysis was done to determine the robustness of the overall effects.

**Results:**

Six studies of 1,768 subjects were included. The primary endpoint that pooled the TVF rate was 8.9% (95% CI 7.5%–10.2%), which comprised the pooled cardiac death (CD) rate (1.5%, 95% CI 0%–3%), myocardial infarction (MI) rate (2.7%, 95% CI 0.4%–5.1%), target vessel revascularization (TVR) (4.8%, 95% CI 2.4%–7.2%), or target lesion revascularization (TLR) (5.2%, 95% CI 4.2%–6.4%), while the secondary endpoint ST was 0.4% (95% CI 0.1%–0.9%). The funnel plots of TVF, CD, TVR, and TLR did not show any serious publication bias, and TVF, TVR, and TLR showed evidence of moderate quality in GRADE assessment. The sensitivity analysis showed that TVF, TLR, and ST exhibited good stability (*I*^2^ = 26.9%, 16.4%, and 35.5%, respectively), while the other endpoints showed moderate instability.

**Conclusion:**

These data indicated that the PzF-nanocoated coronary stents of the Cobra and Catania systems demonstrated good safety and efficacy in clinical application. However, the sample size of patients included in the reports was relatively small, and this meta-analysis will be updated if more studies are published in the future.

**Systematic Review Registration:**

https://www.crd.york.ac.uk/PROSPERO/, identifier: CRD42023398781

## Introduction

It is well recognized that percutaneous transluminal coronary angioplasty and bare metal stent (BMS) have improved the clinical outcomes of coronary heart disease (CHD) dramatically. However, BMS was found to be associated with a high rate of in-stent restenosis (ISR) after percutaneous coronary intervention (PCI), which led to the development of drug-eluting stents (DESs). The new generation of DESs has significantly reduced restenosis by modifying the healing process after stent implantation, attenuating neointimal formation, resulting in a reduction of the incidence of ISR to 5%–10% (1). However, stent thrombosis (ST) is still observed in 2 years of follow-up of DES implantation. A meta-analysis including 10 randomized studies revealed that the incidence of ST did not increase in patients receiving DESs, and the overall rate of ST did not differ significantly among patients receiving sirolimus- or paclitaxel-eluting stents (2). Thus, those with DESs still require a long-term dual antiplatelet therapy (DAPT) for more than 3–6 months, and the duration is usually 12 months (3). Unfortunately, there is a section of the population that cannot receive prolonged DAPT, such as patients with high bleeding risk.

This problem resulted in the development of the Cobra Polyzene-F (PzF) coronary stent (CeloNova BioSiences, San Antonio, TX, USA) (4) and Catania PzF coronary stent (CeloNova Biosciences, Newnan, GA, USA) (5), both of which were designed to address the shortcomings of the DES, especially to decrease ST occurrence. Preclinical studies have shown that these PzF-nanocoated stents exhibited thrombo-resistant, anti-inflammatory, and rapid healing properties in endothelialization (6, 7), and the Cobra PzF stent in a preclinical study suggested a flexible DAPT requirement with potential benefits, especially in patients with high bleeding risk (7). However, whether these two kinds of stents showed consistent efficacy in clinical use was still unclear. Thus, this study was designed to review the efficacy and safety of PzF stents for the treatment of coronary artery lesions. Instead of randomized controlled trials (RCTs), most of the clinical studies investigating PzF-nanocoated coronary stents were designed as prospective, non-randomized, and single-arm studies, with a lack of comparison groups. Therefore, we designed this single-arm meta-analysis to evaluate the safety and efficacy of nanocoated stents in the clinic. This study will help clarify and emphasize the reliability of PzF-nanocoated coronary stents in the clinic and encourage a comparison between nanocoated stents and DESs in further studies.

## Methods

### Registration

This systematic review with the title was registered in PROSPERO (No.398781).

### Data collection

The meta-analysis was designed following PRISMA guidelines. Reports of PzF-nanocoated stents in percutaneous coronary intervention of patients with CHD were searched using MeSH terms and Text Word and collected from PubMed, Embase, and Web of Science with the following terms: [(Polyzene-F) AND (stent)] AND (coronary), or [(Polyzene-F) AND (stent)] AND (coronary) AND (patient), or [(Polyzene-F) AND (stent)] AND (coronary) AND (patient) AND (percutaneous coronary intervention). The three main databases (i.e., Pubmed, Embase, and Web of Science) and other database sources [i.e., China National Knowledge Infrastructure (CNKI) and Google scholar] were searched initially and then screened by reading abstracts and by obtaining full-text articles. Gray literature, especially unpublished manuscripts and ongoing studies, were searched in web engines (i.e., NIH Grants & Funding and the National Natural Science Foundation of China). Records in English and Chinese languages were searched, and the search date covered the period from the time when the database was established to November 2022. Initial literature search was performed by two investigators independently, and the search results were compared and combined, with duplicates removed. Automated tools were not used in this data collection and the following data extraction process.

### Inclusion and exclusion criteria

We aimed to review the efficacy and safety of the two kinds of PzF-nanocoated stents for the treatment of coronary artery lesions. Thus, the inclusion criteria were followed by adopting PICOS principles that included (i) studies among patients with a Polyzene-F nanocoated coronary stent of either the COBRA (4, 8, 9) or the Catania (5, 6) system, (ii) reported target vessel failure (TVF) or major adverse cardiovascular events including CD, MI, target vessel revascularization (TVR), target lesion revascularization (TLR), and ST as the outcomes, (iii) follow-up time during the retrieval between 6 and 12 months, (iv) reported demographical statistics and follow-up, and (v) full-text realization. The exclusion criteria were (i) reported patients who could not receive adjunctive medical therapies such as DAPT or MAPT, (ii) a lack of necessary endpoints as above, or (iii) reviews or case reports.

### Outcome evaluation

The endpoint of the TVF rate (including CD, MI, TVR, and TLR) was set as the primary outcome, and the ST rate was set as the secondary outcome. There was no other variable to be determined in this study. All the pooled data were used to calculate the overall efficacy, and subgroup analysis was performed if there was significant heterogeneity in the overall evaluation.

### Data extraction and synthesis

The full-text articles were obtained after the initial search. Data were extracted from the included studies separately. Any disagreements between the two researchers were resolved by discussion, and a third researcher working as an independent quality evaluator was consulted if no agreement was reached. The information pertaining to data presentation contained parameters such as author, publication date, trial type, objects, demographic data, primary and secondary endpoint rates, and length of follow-up. An assessment of the confounding factors was performed by reading the results and discussion sections in each report. Data presentation and synthesis were carried out by establishing dataset forms and pooled together to be imported to R software. There was no missing summary statistics or data conversions for the included reports in this meta-analysis.

### Appraisal of the quality of evidence

To evaluate the quality of evidence for outcomes, the Grading of Recommendations Assessment, Development and Evaluation (GRADE) profiler software was used for the construction of Summary of Findings (SoF) tables. The quality of evidence was divided into the following four categories: +−very low, ++-- low, +++- moderate, and ++++ high. In GRADE, downgrading of the quality of evidence was based on the risk of bias, inconsistency, indirectness, imprecision, and publication bias, and upgrading in the quality of evidence was based on large effects.

### Statistical analysis

Data from distinct reports were collected and unified to evaluate the safety and efficacy of the PzF-nanocoated coronary stent system. R software (version 3.6.2) was employed to describe the statistical characteristics of data from each article and perform a final single-arm meta-analysis. The positive ratio of each endpoint of counting data was calculated as rates with 95% confidence interval (95% CI), mainly using a random-effects model with the generic inverse variance method, and heterogeneity was examined by using the *Q* test (*p* < 0.1 as heterogeneity) and *I*^2^ statistics (*I*^2^ > 50% as heterogeneity) (10). If there was heterogeneity, then the random-effects model was chosen (11, 12). Moreover, subgroup analysis was performed if there was significant heterogeneity in the overall evaluation of outcomes. The funnel plots with or without the Trim and Fill method and Egger's test were used to check publication bias. The sensitivity analysis was performed to examine the robustness of the overall treatment effects.

## Results

### Overview of studies

**Figure 1:**
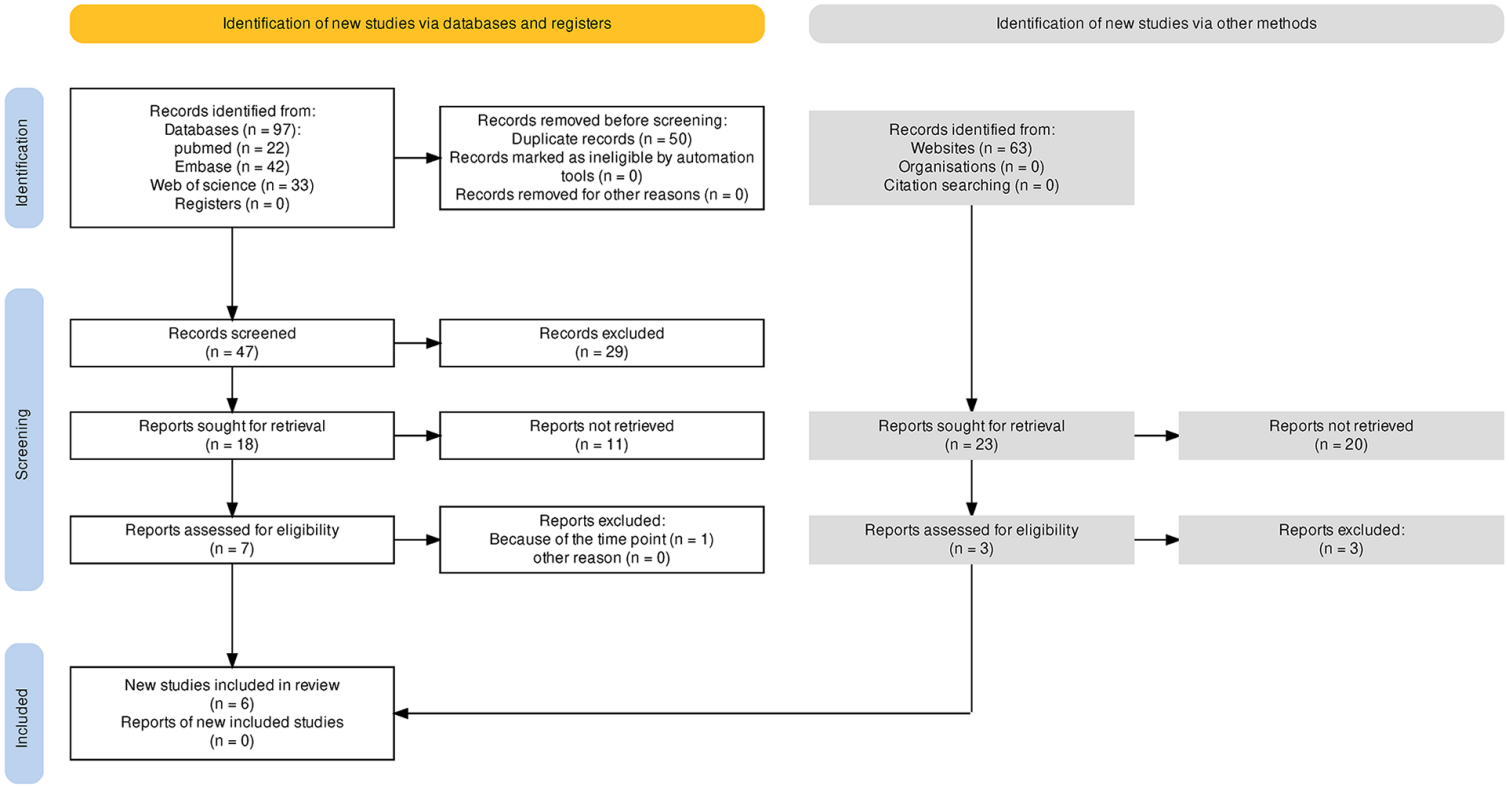
Flow diagram indicates the processs to select and include reports.

First, 97 articles were collected from the three main databases (i.e., PubMed, Embase, and Web of Science), and 63 articles were found in other database sources (i.e., CNKI and Google scholar). After duplicates were removed, 47 articles remained for further screening. A total of 29 records were excluded, which were 16 records of preclinical studies, one record of a case report, three records of study protocols, and nine records of review or correspondence. Then, 18 articles were screened to achieve full text for eligibility assessment. Eleven records were removed and seven studies were included in qualitative synthesis, and then, one study was excluded because of the distinct time point of follow-up. No gray literature, especially unpublished manuscripts and ongoing studies searched in web engines, was included. Finally, six studies with full texts obtained were included for the single-arm meta-analysis (4–6, 8, 9, 13). Figure 1 depicts the search process.

### Characteristics and baseline of the included studies

Overall, this review included six studies with 1,768 subjects, the characteristics and baseline of which are summarized in Table 1. These six studies were comprised of four studies of COBRA (CeloNova Biosciences, San Antonio, TX, USA) (4, 6, 8, 9) and two studies of the Catania (CeloNova BioSciences, Newnan, GA, USA) (5, 13) Polyzene-F nanocoated coronary stent system. The observation periods lasted from 9 to 12 months. The single-arm meta-analysis was performed because of inadequate RCTs. The experimental group in each study was selected, and the patients in this group were treated by using the Polyzene-F nanocoated coronary stent system. The percentage of males involved in this analysis was 72.12 (1,046 from 1,768), and the mean age of all patients involved was 70.14 ± 12.92 years. The percentage of patients with a history of hypertension was 65.95, while patients with a history of dyslipidemia constituted 52.55%. The percentage of patients with angina in clinical presentation was 57.41, while patients with MI in clinical presentation constituted 42.76% (Table 2).

**Table 1:** Description of the characteristics of studies involved in analyses.

#	Author	Patients	Time point (m)	*N*	TVF	CD	MI	TVR	TLR	ST	Nano PZF
1	Cutlip et al. 2017	CHD-PCI	9	296	33	1	20	21	16	0	COBRA
2	Maillard et al. 2020	CHD-PCI	12	940	79	34	44	46	39	12	COBRA
3	Maillard et al. 2017	CHD-PCI	12	100	12	2	5	5	5	0	COBRA
4	Maillard et al. 2019	CHD-HBR-PCI	12	77	2	0	0	0	2	0	COBRA
5	Tamburino et al. 2009	CHD-PCI	12	55	6	0	0	2	6	0	CATANIA
6	Tamburino et al. 2012	CHD-PCI	12	300	25	7	2	24	18	2	CATANIA
Pooled estimate				1,768	157	44	71	98	86	14	

CHD, coronary heart disease; PCI, percutaneous coronary intervention; HBR, high bleeding risk.

**Table 2:** Description of the baseline of studies involved in analyses.

			Sex		Age		History		Clinical presentation	
#	Author	*N*	Male	Female	Mean	SD	Hypertension	Dyslipidemia	Angina	MI
1	Cutlip et al. 2017	296	208	88	66.5	10.3	243	237	249	51
2	Maillard et al. 2020	940	681	259	72.8	13.4	564	396	497	443
3	Maillard et al. 2017	100	71	29	71.4	11	66	63	62	38
4	Maillard et al. 2019	77	45	32	78.7	8.9	57	40	41	36
5	Tamburino et al. 2009	55	41	14	58.6	8.6	33	30	48	6
6	Tamburino et al. 2012	300	229	71	64.9	11.7	203	163	118	182
	Pooled estimate	1,768	1,046	722			963	766	897	574

### Efficacy analysis of the Polyzene-F nanocoated stent in clinical outcomes

**Figure 2:**
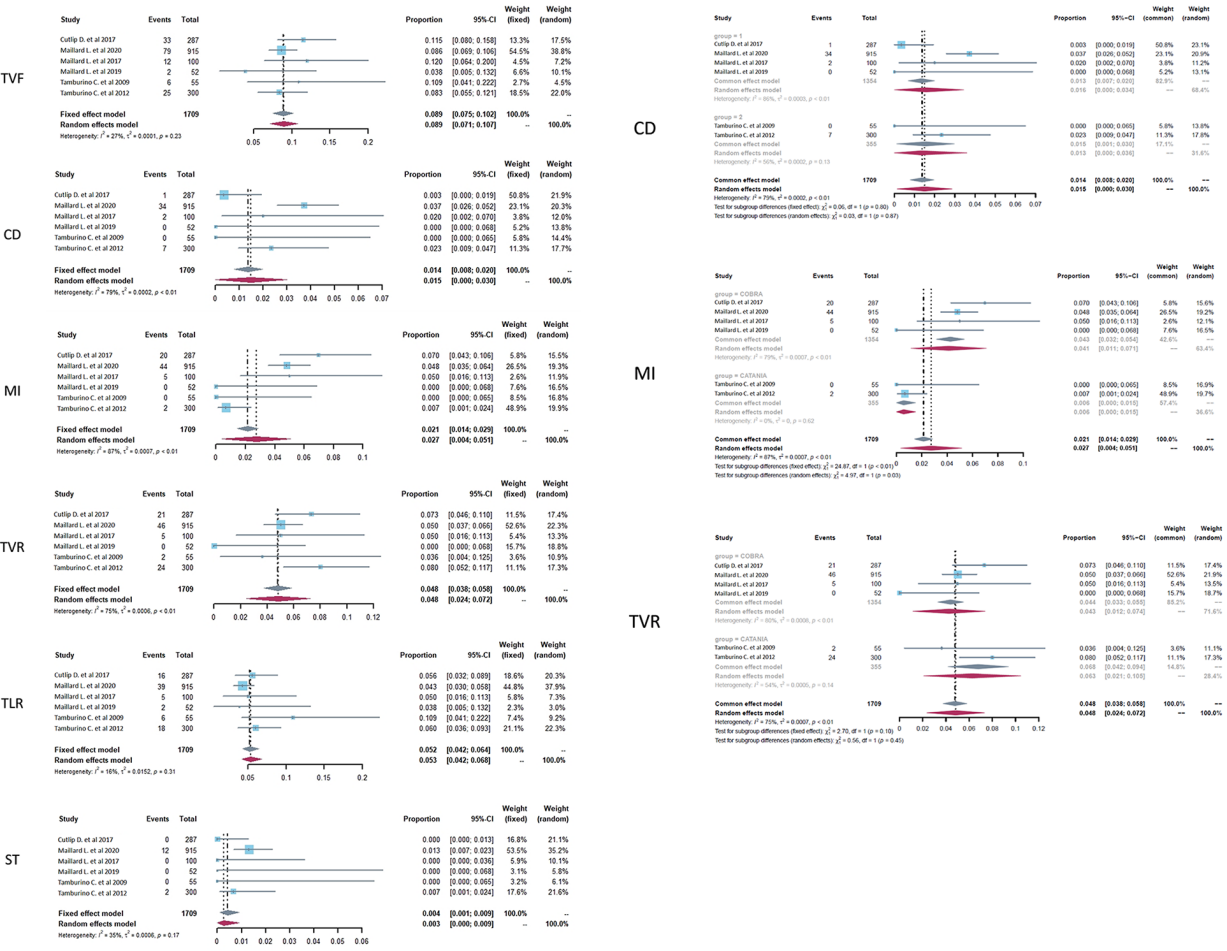
Forest plots of target vessel failure (TVF), cardiac death (CD), myocardial infarction (MI), target vessel revascularization (TVR), target lesion revascularization (TLR), and stent thrombosis (ST) in CHD patients treated with a PzF stent. (Left) The forest plots of selected outcomes with all the data pooled together, (Right) subgroup analysis of the selected outcomes based on the subtype of nanocoated stents.

Clinical events at the endpoint of 9–12 months were pulled together for analysis. The total number of subjects with follow-up was 1709, and forest plots of clinical outcomes are shown in Figure 2, left. The primary endpoint TVF indicated the rate of TVF and defined as a composite of cardiac death (CD), myocardial infarction (MI), or clinically driven target vessel revascularization. TVF occurred in 157 patients, and there was no statistical heterogeneity between studies (*p* = .23, *I*^2^ = 27%). The fixed-effects model indicated that the pooled TVF rate was 8.9%, and the 95% confidence-level boundary ranged from 7.5% to 10.2%. The endpoint CD occurred in 44 patients, and there was heterogeneity (*p* = .01, *I*^2^ = 79%). The random-effects model indicated that the pooled CD rate was 1.5% (95% CI, 0%–3%). The endpoint MI occurred in 71 patients with heterogeneity (*p* < 0.01, *I*^2^ = 87%). Thus, the random-effects model indicated that the pooled MI rate was 2.7% (95% CI, 0.4%–5.1%). Two other endpoints were TVR and TLR, which occurred in 98 patients and 86 patients, respectively. For TVR, heterogeneity was also statistically significant (*p* < 0.01, *I*^2^ = 75%), and the random-effects model indicated that the pooled TVR rate was 4.8% (95% CI, 2.4%–7.2%). For TLR, there was no significant heterogeneity (*p* = .31, *I*^2^ = 16%), and the pooled result was calculated as 5.2% (95% CI, 4.2%–6.4%) in the fixed-effects model. The secondary endpoint ST was defined as the rate of ST occurrence after PCI, and the pooled results showed that ST occurred in 14 patients with slight heterogeneity (*p* = .17, *I*^2^ = 35%). Then, the fixed-effects model showed that the pooled ST rate was 0.4% (95% CI, 0.1%–0.9%).

As there was relatively obvious heterogeneity in CD, MI, and TVR, subgroup analysis was performed on the basis of the subtype of PzF-nanocoated coronary stents (Figure 2, right). The results showed that the main heterogeneity derived from the studies of COBRA stents (CD, *p* < .01, *I*^2^ = 86%; MI, *p* < .01, *I*^2^ = 79%; TVR, *p* < .01, *I*^2^ = 80% in the COBRA subgroup), especially in the outcome parameter of MI (*p* = .62, *I*^2^ = 0 in the CATANIA subgroup).

### Publication bias

**Figure 3:**
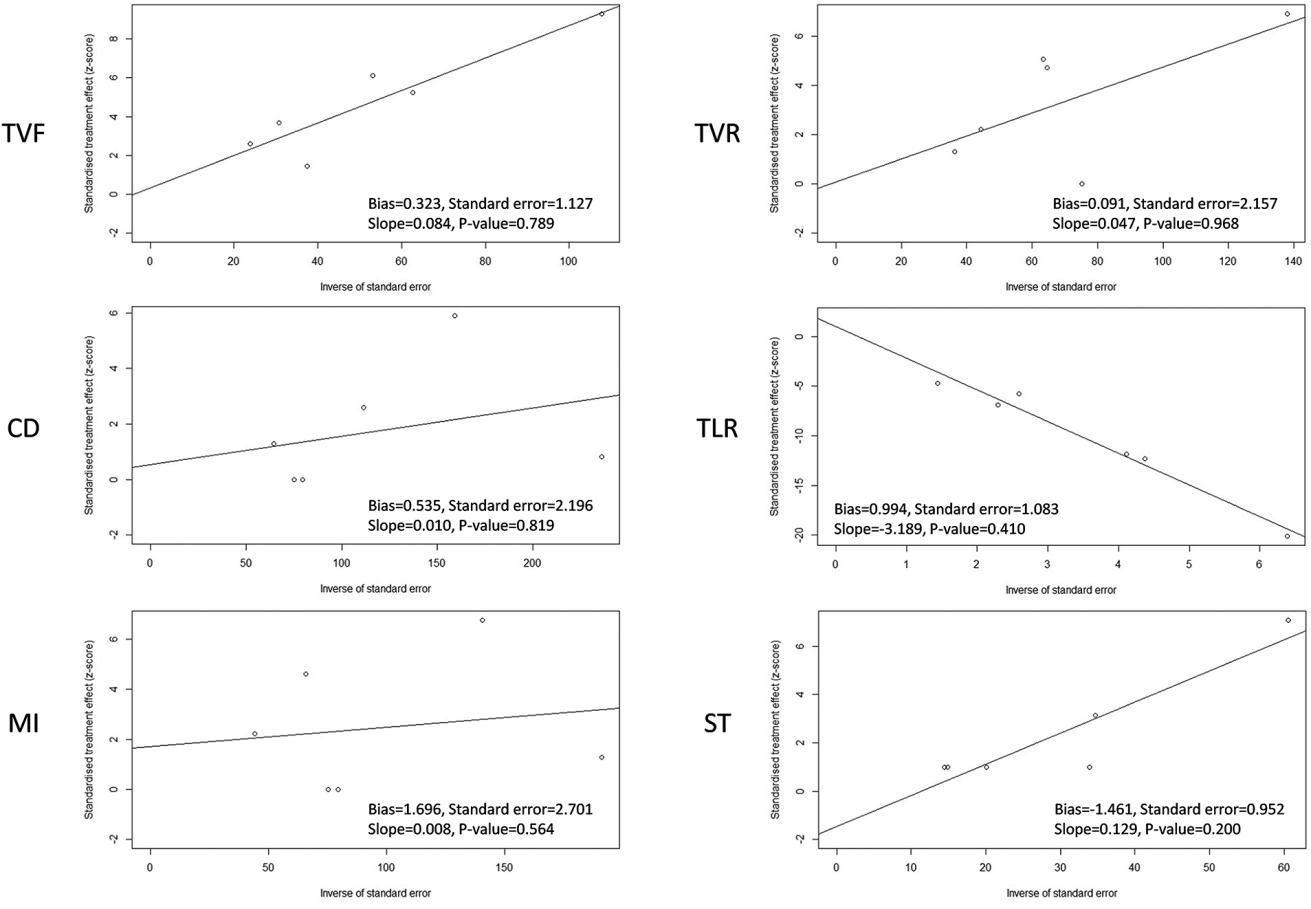
Egger's publication bias plot of target vessel failure (TVF), cardiac death (CD), myocardial infarction (MI), target vessel revascularization (TVR), target lesion revascularization (TLR), and stent thrombosis (ST) in CHD patients treated with a PzF stent.

The funnel plots with or without the Trim and Fill method were used to evaluate the publication bias of the primary and secondary endpoints in CHD patients treated with the Polyzene-F nanocoated coronary stent system (Supplementary Figure S1). Firstly, the funnel plots of the endpoints TVF, CD, TVR, and TLR did not show any serious publication bias, except MI and ST. For MI and ST, the funnel plot indicated a mild publication bias, possibly because of the inadequate number of studies or different parameters in different reports. Moreover, since the key to the funnel plot–based Trim and Fill method lies in estimating the number of missing studies and can be used to control the publication bias, the improved funnel plots using the Trim and Fill method were employed (14–16). The results of the Trim and Fill method showed that three or more studies might be needed to determine the parameters of MI and ST, respectively, which would be able to balance the publication bias in future. Similar results were reported for the bias plots of Egger's publication (Figure 3). As seen in this figure, there were four studies of the ST rate plotted on one side of the line, while there were only two studies plotted on the other side, yielding an imbalanced result. Meanwhile, the intercepts of the *Y*-axis of MI and ST were 1.697 and −1.461, respectively, which were at a great distance from 0 compared with any other endpoint indicators, suggesting the probable existance of publication bias (Supplementary Table S1). For the other endpoints, barring MI and ST, the studies were plotted almost in a balance fashion, which indicated minimal publication bias.

### Assessment of the quality of evidence

To evaluate the quality of evidence for outcomes, the SoF table was constructed by using the GRADE profiler software. The summary of the overall evidence for each outcome according to the GRADE method is presented in Table 3. The results showed that the quality of evidence was moderate for TVF, TVR, and TLR and low for CD and ST, while the quality of evidence was very low for MI. These results could be mainly attributed to the type of the included clinical trials, which was a single-arm clinical trial design, which increased the risk of bias, especially selection bias and performance bias.

**Table 3:** SoF table created by using GRADE software.

Outcomes	Illustrative comparative risks^1^ (95% CI)		Relative effect (95% CI)	No of participants (studies)	Quality of the evidence (GRADE)
	Assumed risk	Corresponding risk			
	Control	ISR			
TVF Follow-up: 12 months	Study population		Risk difference 0.09 (0.08–0.1)	1,768 (6 studies)	⊕⊕⊕⊝ Moderate^2^
	0 per 1,000	89 per 1,000 (75–102)			
CD Follow-up: 12 months	Study population		Risk difference 0.01 (0.01–0.02)	1,768 (6 studies)	⊕⊕⊝⊝ Low^2^
	0 per 1,000	15 per 1,000 (0–30)			
MI Follow-up: 12 months	Study population		Risk difference 0.02 (0.01–0.03)	1,768 (6 studies)	⊕⊝⊝⊝ Very low^2^
	0 per 1,000	27 per 1,000 (4–51)			
TVR Follow-up: 12 months	Study population		Risk difference 0.05 (0.04–0.06)	1,768 (6 studies)	⊕⊕⊕⊝ Moderate^2^
	0 per 1,000	48 per 1,000 (38–58)			
TLR Follow-up: 12 months	Study population		Risk difference 0.05 (0.04–0.06)	1,768 (6 studies)	⊕⊕⊕⊝ Moderate^2^
	0 per 1,000	52 per 1,000 (42–64)			
ST Follow-up: 12 months	Study population		Risk difference 0.00 (0–0.01)	1,768 (6 studies)	⊕⊕⊝⊝ Low^2^
	0 per 1,000	4 per 1,000 (1–9)			
GRADE Working Group grades of evidence *High quality:* Further research is highly unlikely to change our confidence in the estimate of effect. *Moderate quality:* Further research is likely to have an important impact on our confidence in the estimate of effect and may change the estimate. *Low quality:* Further research is highly likely to have an important impact on our confidence in the estimate of effect and is likely to change the estimate. *Very low quality:* We are very uncertain about the estimate.					

^1^ The basis for the *assumed risk* (e.g. the median control group risk across studies) is provided as follows. The *corresponding risk* (and its 95% confidence interval) is based on the assumed risk in the comparison group and the *relative effect* of the intervention (and its 95% CI). CI, confidence interval.

^2^ The quality of evidence for the outcomes included target vessel failure (TVF), cardiac death (CD), myocardial infarction (MI), target vessel revascularization (TVR), target lesion revascularization (TLR) and stent thrombosis (ST).

### Sensitivity analysis

**Figure 4:**
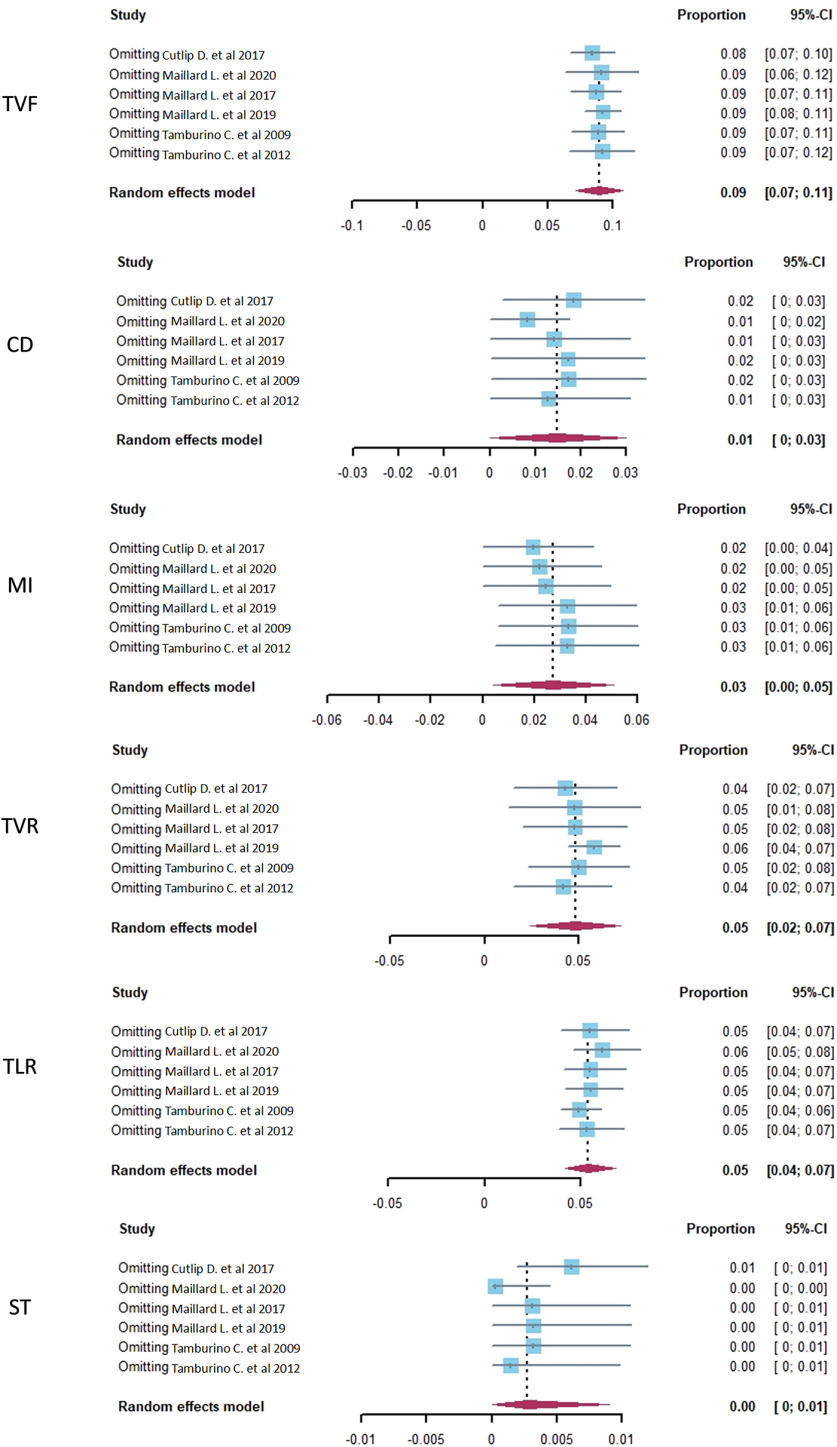
The sensitivity analysis of target vessel failure (TVF), cardiac death (CD), myocardial infarction (MI), target vessel revascularization (TVR), target lesion revascularization (TLR), and stent thrombosis (ST) in CHD patients treated with a PzF stent.

A sensitivity analysis of the Polyzene-F nanocoated coronary stent system was conducted by eliminating the included studies one by one to check the endpoints of TVF, CD, MI, TVR, TLR, and ST, and the pooled estimates results are listed in Table 4. The results showed that TVF, TLR, and ST exhibited relatively good stability (*I*^2^ = 26.9%, 16.4% and 35.5%, respectively), while the other endpoints, CD, MI, and TVR showed moderate instability (*I*^2^ = 78.5%, 87.3%, and 75.1%, respectively). The forest plots of the sensitivity analysis demonstrated the weight of each study in causing instability to each endpoint (Figure 4).

**Table 4:** The sensitivity analysis result of target vessel failure (TVF), cardiac death (CD), myocardial infarction (MI), target vessel revascularization (TVR), target lesion revascularization (TLR), and stent thrombosis (ST) in coronary heart disease patients treated with a Polyzene-F stent; pooled estimate in the random-effects model (related to Figure 4).

#	Indicator	Proportion	95% CI	tau^2^	*I*^2^ (%)
1	TVF	0.0893	[0.0712; 0.1074]	0.0001	26.9
2	CD	0.0148	[−0.0002; 0.0298]	0.0002	78.5
3	MI	0.0274	[0.0000; 0.0507]	0.0007	87.3
4	TVR	0.0484	[0.0243; 0.0725]	0.0006	75.1
5	TLR	0.0534	[0.0420; 0.0679]	0.0152	16.4
6	ST	0.0027	[0.0000; 0.0091]	0.0006	35.5

## Discussion

This study attempted to review the safety and effectiveness of the PzF-nanocoated coronary stent and analyzed the clinical studies of both the COBRA and Catania coronary stent systems in a single-arm meta-analysis. The primary endpoint indicator of the pooled TVF rate was 8.9%, containing the pooled CD rate (1.5%), MI rate (2.7%), and TVR and TLR (4.8% and 5.2%, respectively), while the secondary endpoint ST was 0.4% in our pooled data. In the literature, DESs were always used to make a comparison with a nanocoated stent (4), and our data revealed that the PzF-nanocoated stent achieved excellent results, both in TVF and in ST.

As is known, DESs have reduced the risks of restenosis and TLR after PCI. However, delayed endothelialization, stent malapposition, and inflammatory reactions may also occur (17). Thus, those with DESs still require a long-term administration of DAPT according to current guidelines, but this results in high bleeding risk and high cost. Unfortunately, some patients cannot receive prolonged DAPT because of the increased risk of developing stent thrombosis (18). To address these shortcomings of DESs, the development of biologically based platforms is encouraged.

Both the Cobra PzF (CeloNova BioSiences, San Antonio, TX, USA) and the Catania PzF coronary stent (CeloNova Biosciences, Newnan, GA, USA) were designed as stents with a surface-coated thin nanolayer of PzF. The nanolayer surface of PzF is an approximately 40- nm-thick coating of an inorganic rubber that has been shown to be associated with the preferential adsorption of albumin, low distortion of protein structures, and low platelet adhesion and activation (19). The nanolayer PzF exhibits superior properties such as thromboresistance (20), confirmed by preclinical studies during follow-ups (9). Thus, Cobra PzF was approved in 2017 (7).

As the DES becomes the main stent for PCI, and BMS is rarely used in clinical practice nowadays, a comparison of TVF and ST in BMS vs. PzF-nanocoated stent becomes unnecessary. However, the DES has impaired endothelialization and late ST (19), and studies showed that the administration of DAPT after PCI usually prolonged to 6–12 months (21), and this period can be as short as 3 months in newer-generation DESs (22). In this study, the low ST rate of 0.4% supports published reports that the PzF-nanocoated stent showed a markedly reduced thrombogenicity. These data indicated that the PzF-nanocoated coronary stents of the COBRA and Catania systems demonstrated good safety and efficacy in clinical application.

## Conclusion

In conclusion, our review provides evidence that the nanocoated stent may be another choice in PCI with a low stent thrombosis formation rate. This indicates that the PzF-nanocoated coronary stents of the COBRA and Catania systems demonstrate good safety and efficacy in clinical application.

## Limitation and perspective

As the amount of included reports and the total objective size were relatively small in our single-arm meta-analysis, publication bias analysis and sensitivity analysis were performed, which yielded relatively robust results. Moreover, there was a relatively obvious heterogeneity in some outcome parameters, and subgroup analysis showed that the main heterogeneity derived from the studies of COBRA stents. The main limitation of this study is the lack of comparison studies such as an RCT clinical trial design, and the publication bias analysis may produce more accurate results when more studies are included. Finally, the sample size of patients included in the reports is relatively small, and this meta-analysis will be updated if more studies are published in this field in the future.

## Data Availability

The raw data supporting the conclusions of this article will be made available by the authors without undue reservation.
