# Supplementary material for: Evaluation of the safety and efficacy of a Polyzene-F nanocoated coronary stent system: A systematic review and single-arm meta-analysis
Source: Front Cardiovasc Med. 2023 Mar 16;10:1095794. doi: 10.3389/fcvm.2023.1095794 (PMC10063189; doi:10.3389/fcvm.2023.1095794)
Supplement: Supplementary file 1 [file Datasheet1.pdf]

Supplementary information

Supplementary Figure 1

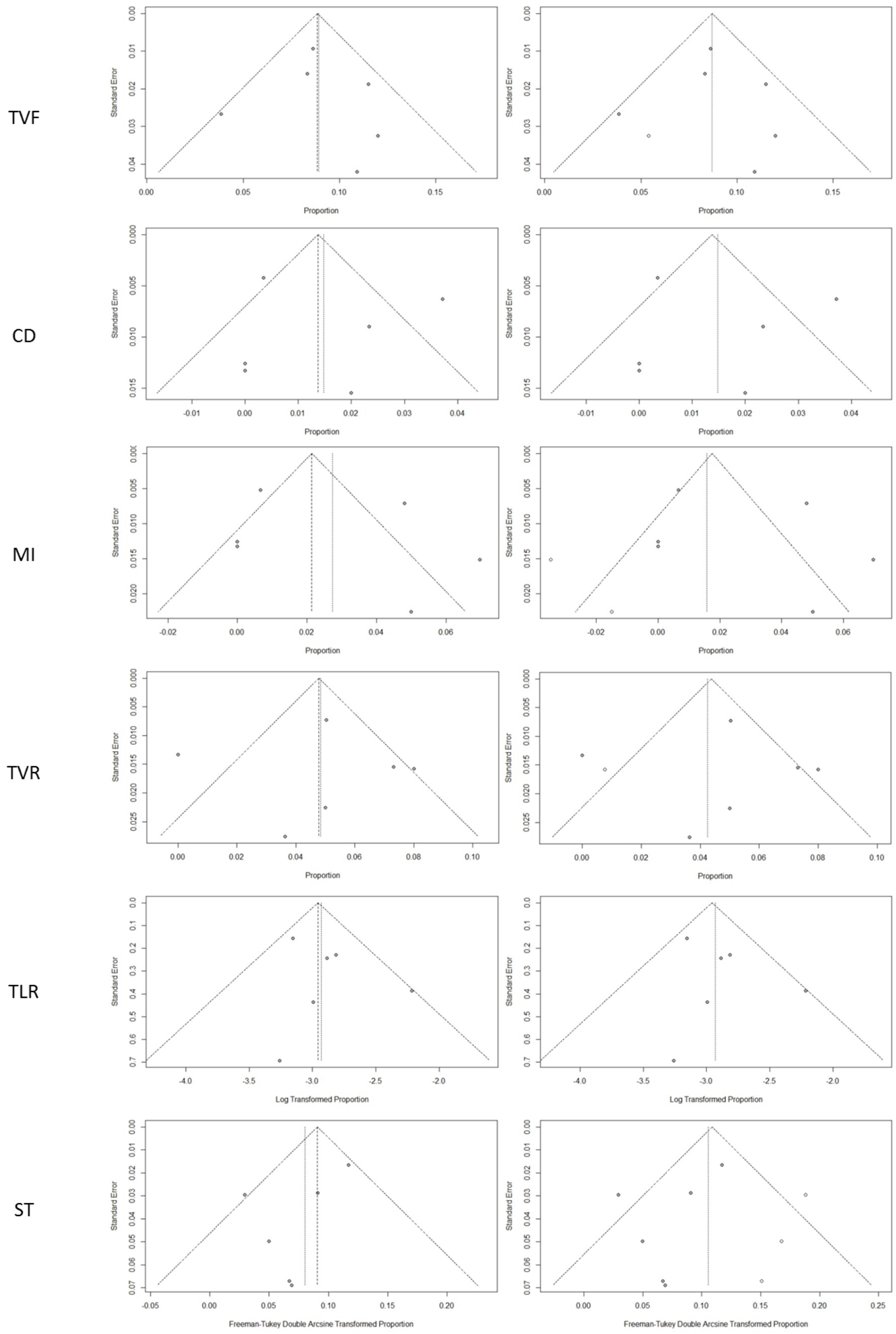

Supplementary Table 1. The Egger's test result for publication bias plot of target vessel failure (TVF), cardiac death (CD), myocardial infarction (MI), target vessel revascularization (TVR), target lesson revascularization (TLR) and stent thrombosis (ST) in coronary heart disease patients treated with Polyzene-F stent (related to Figure 3).

#	Indicator	Bias	Se.bias	Slope	t	P
1	TVF	0.323	1.127	0.084	0.287	0.789
2	CD	0.535	2.196	0.010	0.244	0.819
3	MI	1.696	2.701	0.008	0.628	0.564
4	TVR	0.091	2.157	0.047	0.042	0.968
5	TLR	0.994	1.083	-3.189	0.918	0.410
6	ST	-1.461	0.952	0.129	-1.534	0.200
